# Indoor Mapping with Entertainment Devices: Evaluating the Impact of Different Mapping Strategies for Microsoft HoloLens 2 and Apple iPhone 14 Pro

**DOI:** 10.3390/s24041062

**Published:** 2024-02-06

**Authors:** Jiwei Hou, Patrick Hübner, Jakob Schmidt, Dorota Iwaszczuk

**Affiliations:** 1 Remote Sensing and Image Analysis, Department of Civil and Environmental Engineering, Technical University of Darmstadt, 64287 Darmstadt, Germany; jiwei.hou@tu-darmstadt.de (J.H.); dorota.iwaszczuk@tu-darmstadt.de (D.I.); 2 Geodetic Measurement Systems and Sensor Technology, Department of Civil and Environmental Engineering, Technical University of Darmstadt, 64287 Darmstadt, Germany; jakob.schmidt1@tu-darmstadt.de

**Keywords:** indoor mapping, entertainment devices, HoloLens 2, iPhone 14 Pro, mapping strategies, augmented reality (AR)

## Abstract

Due to their low cost and portability, using entertainment devices for indoor mapping applications has become a hot research topic. However, the impact of user behavior on indoor mapping evaluation with entertainment devices is often overlooked in previous studies. This article aims to assess the indoor mapping performance of entertainment devices under different mapping strategies. We chose two entertainment devices, the HoloLens 2 and iPhone 14 Pro, for our evaluation work. Based on our previous mapping experience and user habits, we defined four simplified indoor mapping strategies: straight-forward mapping (SFM), left–right alternating mapping (LRAM), round-trip straight-forward mapping (RT-SFM), and round-trip left–right alternating mapping (RT-LRAM). First, we acquired triangle mesh data under each strategy with the HoloLens 2 and iPhone 14 Pro. Then, we compared the changes in data completeness and accuracy between the different devices and indoor mapping applications. Our findings show that compared to the iPhone 14 Pro, the triangle mesh accuracy acquired by the HoloLens 2 has more stable performance under different strategies. Notably, the triangle mesh data acquired by the HoloLens 2 under the RT-LRAM strategy can effectively compensate for missing wall and floor surfaces, mainly caused by furniture occlusion and the low frame rate of the depth-sensing camera. However, the iPhone 14 Pro is more efficient in terms of mapping completeness and can acquire a complete triangle mesh more quickly than the HoloLens 2. In summary, choosing an entertainment device for indoor mapping requires a combination of specific needs and scenes. If accuracy and stability are important, the HoloLens 2 is more suitable; if efficiency and completeness are important, the iPhone 14 Pro is better.

## 1. Introduction

Over the past few decades, with the development of laser scanning technology, particularly the introduction of terrestrial laser scanning (TLS) technology in the 1990s [1,2], great progress has been made in indoor and outdoor mapping. TLS is capable of generating high-density point cloud data that accurately captures the three-dimensional (3D) structure of the scanning space [3]. Since then, TLS has been further expanded with the maturity of mobile laser scanning (MLS) technology and simultaneous localization and mapping (SLAM) [4,5]. MLS systems incorporate laser scanners on mobile platforms such as vehicles [6,7,8], drones [9,10,11], and backpacks [12,13], allowing them to capture data in indoor and outdoor environments while moving. At the same time, the use of SLAM technology allows the MLS system to locate and build maps in real time in unknown environments, no longer limited by the mapping scope of static sensors. Nowadays, MLS systems are widely used in areas such as indoor and outdoor mapping [14,15], forest survey [16,17], and cultural heritage protection [18,19].

However, with the development of computer vision, sensor technologies, and 3D modelling tools, researchers are increasingly interested in the possibilities of employing entertainment devices like consumer-grade depth cameras [20,21,22,23], smartphones [24,25,26], and augmented reality (AR) headset devices [27,28,29,30] for indoor mapping. While these entertainment devices are not specifically designed for indoor mapping, they are getting a lot of attention in this field because of their advanced sensing technology and high-performance computing, as well as their high degree of portability, which gives them great potential for indoor mapping. In comparison to the expensive industry-grade TLS and MLS mapping systems, the mapping approach using low-cost entertainment devices enables more people to participate in indoor mapping and offers great potential for applications such as indoor navigation, building information modeling (BIM), and facility management.

In 2010, Microsoft introduced the first-generation Kinect [31], a depth camera based on structured light technology for depth perception. Although Kinect was originally marketed as an entertainment add-on for Xbox consoles, with its affordable depth-sensing capabilities, it has surprisingly become a catalyst for innovation across various domains. Among the groundbreaking advancements, KinectFusion [20] stood out as a technology that used the Kinect’s depth-sensing abilities to generate comprehensive 3D maps of indoor environments. This innovation enabled users to scan and reconstruct real-world spaces in real-time with a depth camera [20,21,32], opening up new uses beyond gaming. Over time, next-generation depth cameras like Intel RealSense [33,34] and Azure Kinect [35] further enhanced the ability to acquire accurate depth information in indoor environments.

For indoor mapping with smartphones, Google’s Project Tango [36] was an early experimental project in 2014 aimed at improving mobile devices’ spatial perception of the environment by integrating depth perception technology on Android smart devices. One of the goals of the project was to achieve 3D indoor mapping on mobile devices [37,38]. While Project Tango ended with the appearance of ARCore [39], its contribution to depth perception technology and indoor mapping has influenced the development of subsequent projects and technologies. In 2017, the emergence of AR frameworks like ARCore and ARKit [40] has significantly simplified the development of smartphone-based AR applications. In the work of Hasler et al. [25], they developed two smartphone indoor mapping applications based on the AR framework in their study. By evaluating them in complex indoor environments, their work demonstrated the great potential of smartphone-based 3D indoor mapping, with applications achieving an absolute 3D accuracy of around 1% of walking distance in indoor environments, and sub-centimeter local 3D accuracy.

To advance AR and depth perception technologies, in October 2020, Apple released its first mobile smart devices equipped with light detection and ranging (LiDAR) sensors. The main purpose of these LiDAR sensors is to improve camera focus speed in low-light environments and provide more accurate depth perception. While other devices such as the Samsung Galaxy S23 Ultra and Huawei Mate 60 Pro also have advanced camera systems and processing capabilities, they lack a dedicated depth sensor like LiDAR, which is a significant advantage for detailed indoor mapping tasks.

For 3D mapping with LiDAR-equipped Apple smart devices, Díaz-Vilariño et al. [24] evaluated the potential of Apple’s smart devices for indoor and outdoor 3D mapping applications using a LiDAR-equipped iPad Pro. Their study indicated that Apple smart devices are not suitable for mapping large environments and emphasized the importance of acquisition planning to avoid complex large trajectories. In addition, Jakovljević et al. [41] investigated the performance of rapid indoor 3D point cloud acquisition using an iPhone 13 Pro equipped with LiDAR. Their results show that the iPhone 13 Pro is able to provide accurate and stable point clouds on flat or curved surfaces with an average absolute distance of 9 cm compared to high-precision TLS data. The iPhone 13 Pro performs well in capturing detailed scenes, but noise increases on homogeneous surfaces and direct sunlight decreases the accuracy of the point cloud.

Along with the frameworks for AR applications that run on smartphones, there are also AR headset devices. To achieve a more stable immersive AR experience, AR headset devices often integrate advanced tracking and mapping algorithms. These algorithms help align virtual content with the real world with a high degree of accuracy, allowing users to interact with virtual objects and navigate in the real world. For indoor mapping, AR headset devices such as the Microsoft HoloLens [42], Magic Leap [43], and Google Glass [44] can be used. 

There are some notable evaluation studies of indoor mapping using AR headset devices, for example, Hübner et al. [28,29] and Khoshelham et al. [30] first quantitatively evaluated the indoor mapping capabilities of Microsoft HoloLens (Version 1) regarding the context of indoor building geometry. Hübner et al. [28] also investigated the depth sensing and tracking capabilities of the HoloLens in their study. By comparing with TLS ground truth data, Hübner et al. [28,29] found that changes in room conditions may affect the final indoor mapping quality of the HoloLens, e.g., the presence or absence of furniture in the indoor environment. Thus, when mapping in unfurnished or less textured indoor environments, the transition area between different rooms may be a weak point in the mapping of the HoloLens, which is prone to large errors that affect the accuracy of the overall measurement data. Weinmann et al. [45,46] demonstrated that the data acquired by the HoloLens is accurate enough to be used to reconstruct semantically rich and topologically correct indoor scene models. Jäger et al. [47] in their study performed neural radiance fields (NeRFs) [48,49,50] 3D reconstruction using internal camera poses and images provided by the HoloLens, and revealed the potential of combining the HoloLens with NeRFs for highly detailed, colored, mobile 3D mapping. Teruggi et al. [51] tested the mapping capabilities of the HoloLens 2 in complex monumental spaces. Aside from the study around the HoloLens, Demirkan et al. [52] conducted a study evaluating the performance of AR-assisted navigation in real underground mine conditions. Utilizing the embedded spatial mapping algorithm on the Magic Leap One, they found that AR-assisted navigation significantly facilitated evacuation, demonstrating effectiveness in supporting search and rescue efforts in challenging underground environments. Li et al. [53] implemented VisioMap using Google Glass. The system uses sparse photograph samples at eye-level to reconstruct 3D indoor scenes, and its lightweight approach, based on geometric features rather than image pixels, enables accurate localization without the need for dense fingerprints or points of interest, confirming its usability as a prototype for natural indoor localization.

In addition to the entertainment devices described above, Holzwarth et al. [54] conducted a comparative analysis of motion tracking for entertainment virtual reality (VR) devices, specifically SteamVR Tracking and Oculus Insight, in a medium room scale setup, using the Oculus Quest 2 [55] and HTC Vive Tracker [56], revealing the superior accuracy and precision in the height and position tracking of the Oculus Quest 2, making it a viable choice for various applications, including indoor mapping. Of course, there are also studies based on classical photogrammetry and computer vision algorithms for indoor mapping using cameras with different lenses [57,58,59], this approach generally requires using software based on Structure-from-Motion (SfM) [60,61] and Multi-View Stereo (MVS) [62] for 3D reconstruction, like COLMAP [63].

Some existing studies have evaluated the accuracy of entertainment devices for indoor mapping applications at the level of built-in depth sensors, tracking algorithms, and indoor mapping capabilities. However, to the best of our knowledge, there is currently no in-depth study on the performance of entertainment devices in indoor mapping under different acquisition behaviors or strategies, such as directly forward mapping, alternatively left and right mapping, and round-trip mapping while holding or wearing these devices. Users with different skill levels and experience may adopt different scanning methods and habits when using these entertainment devices for mapping, which is somewhat random in nature. Due to the lack of uniform norms and standards, the impact of user behavior on the outcome of indoor mapping with these devices is notably significant.

Although some studies have hinted at the importance of the data collection strategy, a thorough investigation into its effects is still lacking. This is crucial because different mapping strategies can significantly influence the results, as Askar et al. [64] have demonstrated through their in-depth study using the iPhone 13 Pro for indoor mapping. They divided the entire room into two separate sections and repeated the scanning process multiple times to achieve optimal results. Additionally, Hübner et al. [28,29] found that operators using the HoloLens could improve indoor mapping accuracy by looking back at mapped spaces when entering new areas that had not been scanned, especially when in transitional spaces such as doors. This observation also highlights the importance of operator behavior and suggests the need for further research into its impact on indoor mapping accuracy.

Therefore, the aim of this study is to evaluate the potential impact that different mapping strategies may have on the final results when using entertainment devices for indoor mapping. In this study, we chose the HoloLens 2 and iPhone 14 Pro as our experimental devices. We hope that our work will provide a reference for future professionals and non-professionals involved in indoor mapping applications on how to optimize mapping strategies in order to make full use of the device’s capabilities and more rapidly and accurately acquire the best mapping data.

In the following, we outline the experimental process in Section 2. Subsequently, Section 3 presents the results, followed by an in-depth discussion in Section 4. Finally, Section 5 concludes with our remarks.

## 2. Materials and Methods

**Figure 1:**
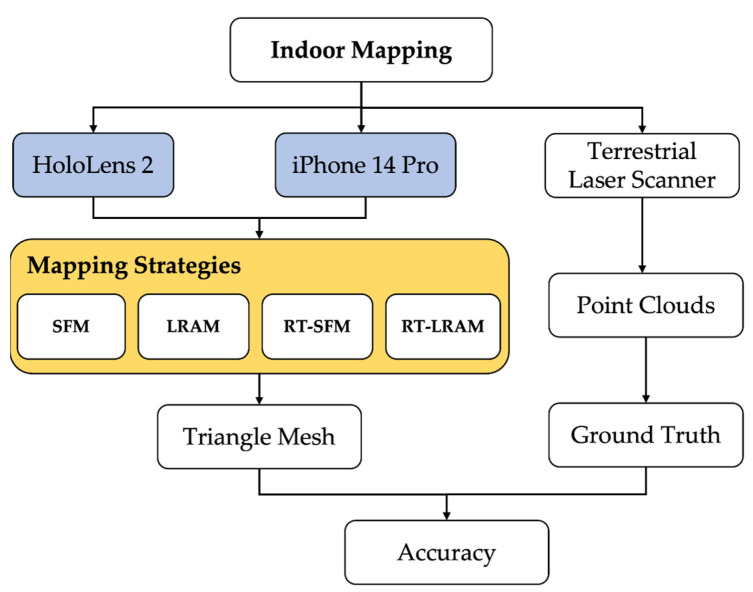
Schematic overview of the evaluation of the impact of different strategies on indoor mapping accuracy using the HoloLens 2 and iPhone 14 Pro, respectively.

In this section, we provide a detailed overview of the experimental process, which aims to assess the impact of different mapping strategies on indoor mapping accuracy using the HoloLens 2 and iPhone 14 Pro. First, in Section 2.1, we introduce the selected experimental space, ensuring that the experimental environment exhibits sufficient representativeness. Subsequently, in Section 2.2, we define the various mapping strategies used in our study. Finally, in Section 2.3, we comprehensively describe a series of evaluation procedures employed throughout this study. A schematic overview of the whole evaluation process is shown in Figure 1. 

### 2.1. Experimental Space

**Figure 2:**
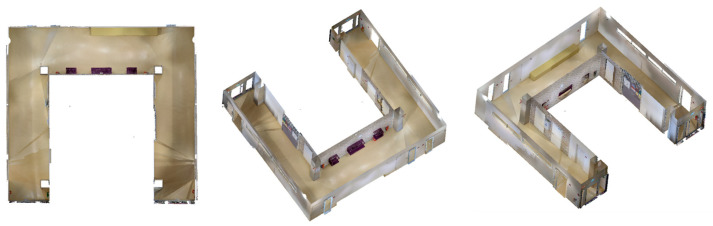
The experimental space. The ceiling is removed for better visibility.

Due to the complexity and variability of indoor environments, it is difficult to choose a representative space that is universally applicable. When selecting the experimental environment, based on our prior knowledge, we mainly considered three criteria: simplicity of the space structure, stability of the lighting conditions and applicability of the equipment. Finally, we chose a corridor inside a building as the experimental site, as shown in Figure 2.

The relatively simple layout of the experimental space helps to minimize potential interfering factors such as furniture, curtains, and plants, allowing for better control of the experimental conditions and resulting in more interpretable results.

Compared to rooms with rich furniture arrangements, the central part of this corridor has only a small amount of furniture blocking the walls. This arrangement not only creates more conducive conditions for the propagation of signals from sensors such as LiDAR and vision sensors, making image acquisition easier, but also facilitates our assessment of the impact of a small amount of furniture occlusion on different indoor mapping strategies.

Additionally, the illumination in the corridor is relatively stable, which minimizes the potential interference caused by lighting condition changes during sensor signal acquisition. Moreover, with a total length of approximately 30 m and a width ranging from 2 to 3 m, the corridor offers a moderate scale. Especially the width of the space is well within the maximum depth perception of the HoloLens 2 and iPhone 14 Pro, and this makes the corridor particularly suitable for the evaluation of different indoor mapping strategies with the HoloLens 2 or iPhone 14 Pro.

### 2.2. Strategies Description

During the process of indoor mapping using entertainment devices such as the Microsoft HoloLens 2 or iPhone 14 Pro, user behavior often exhibits significant randomness, and the mapping process lacks a uniform specification and is challenging to classify. According to our experience, specific patterns of walking and looking at the surrounding environment are developed habitually by the user when frequently using these devices for indoor mapping tasks.

**Figure 3:**
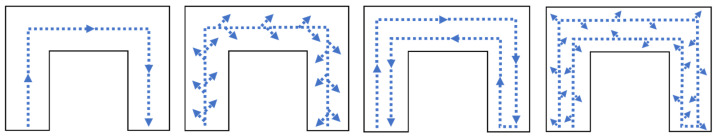
Four different mapping strategies in our research, from left to right are SFM, LRAM, RT-SFM and RT-LRAM.

In general indoor scenarios, ceilings are often obstructed by luminaires and decorative materials, making it challenging to scan ceilings completely with the HoloLens 2 or iPhone 14 Pro. In addition, ceilings are usually removed in studies to enhance visibility. Therefore, in this study, we primarily focus on the surrounding walls and the floor within indoor environments when defining mapping strategies. Based on our experience with indoor mapping research, we make a first step towards classifying mapping strategies in user behavior during indoor mapping by defining four simplified mapping strategies, as shown in Figure 3, including:

- Straight-Forward Mapping (SFM): Devices are oriented directly ahead.
- Left–Right Alternating Mapping (LRAM): Devices oscillate horizontally while mapping.
- Round-Trip Straight-Forward Mapping (RT-SFM): In the experimental environment, the user walks a round trip while the devices are oriented directly ahead during the mapping process.
- Round-Trip Left–Right Alternating Mapping (RT-LRAM): Similar to RT-SFM, the user walks a round trip while the devices oscillate horizontally while mapping.

We conducted a series of indoor mapping experiments with these four strategies using the HoloLens 2 and iPhone 14 Pro, respectively. 

### 2.3. Evaluation Method

To evaluate the influence of different mapping strategies on indoor mapping using entertainment devices, we conducted a series of experiments with the Microsoft HoloLens 2 and iPhone 14 Pro, respectively. 

**Figure 4:**
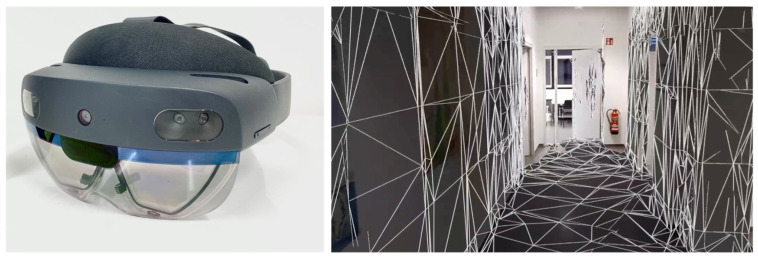
The HoloLens 2 used in our research and the user’s view during mapping.

The HoloLens 2 used in this study is shown in Figure 4. For HoloLens indoor mapping, Hübner et al. [29], in their research, utilized the commercial app SpaceCatcher to generate triangle mesh data in their HoloLens 1 indoor mapping evaluation work. Although we intended to use the same app for data collection, we found the SpaceCatcher has stopped being updated and is incompatible with the HoloLens 2.

To the best of our knowledge, currently there is no specific off-the-shelf HoloLens 2 app for triangle mesh data collection. However, this gives us the opportunity to evaluate only the built-in algorithms of the HoloLens 2. Unlike other software applications that may include their own tracking and mapping algorithms or optimize the built-in algorithms, our focus is solely on the performance of the built-in algorithms of the HoloLens 2. 

Through the open-source HoloLens 2 Sensor Streaming (hl2ss) Library [65], which is based on the officially provided Application Programming Interface (API), we can obtain the raw tracking and mapping data directly from the HoloLens 2 without any post-processing or algorithm modifications. This approach ensures that our findings are solely attributed to the device’s capabilities and not influenced by external software applications.

**Figure 5:**
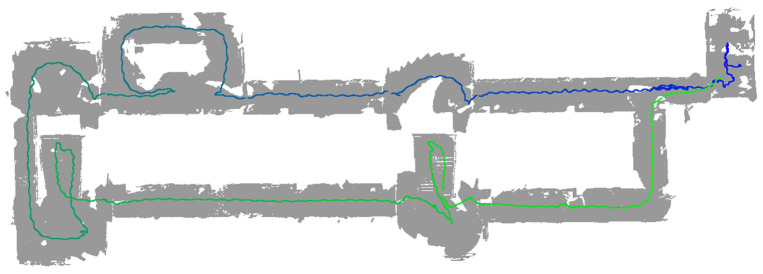
HoloLens 2 indoor mapping test between two floors of a building. The trajectory starts at blue and ends at green.

Our self-developed indoor mapping script has been tested and is now able to generate large scale 3D triangle mesh data with associated mapping trajectories, as shown in Figure 5. Compared to the method of exporting OBJ format triangle meshes data directly from the web device portal, we were able to configure our mapping process more flexibly to meet our desired resolution requirements and to record movement trajectories. In this study, we set the maximum triangles per cubic meter to 10,000. This approach provides greater flexibility and extensibility, allowing us to do further in-depth research with the HoloLens 2. Moreover, we also utilized the augmented reality (AR) feature of the HoloLens 2 to add the virtual triangle meshes in the user’s field of view during mapping, so that we can have a better immersive experience during the mapping process, as shown in Figure 4.

Unlike the HoloLens 2, for indoor mapping with the iPhone 14 Pro, we can directly download off-the-shelf apps from the App Store for our evaluation work. Due to the difference in algorithms and parameter configurations, indoor mapping accuracy may be different among applications. Although we were unable to fully evaluate all mapping applications in the App Store, with reference to [24,64,66,67], we selected PIX4DCatch and 3D Scanner as representatives and conducted a comparison experiment. In this way, we explored whether variations in mapping accuracy on the iPhone 14 Pro exhibit consistency when employing different mapping strategies for indoor mapping using different applications. Subsequently, we conducted a comparative analysis between the mapping results of the iPhone 14 Pro and HoloLens 2 to investigate if differences in mapping accuracy remained consistent across various mapping devices.

**Figure 6:**
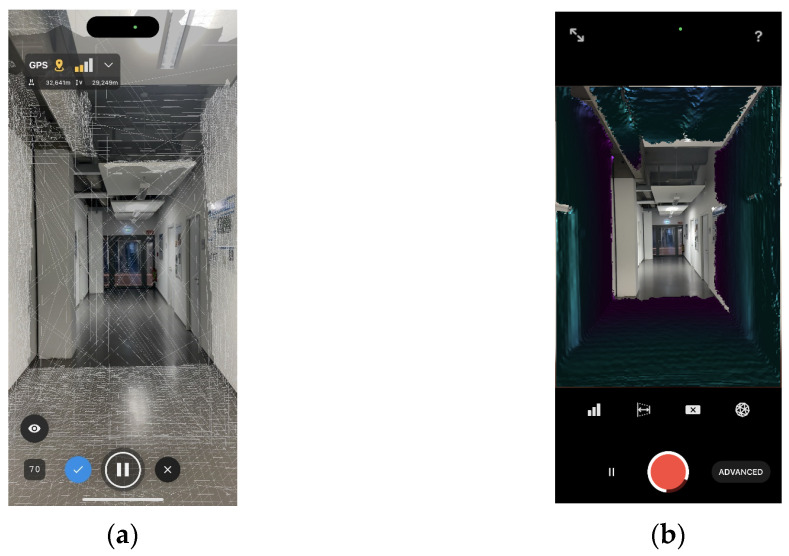
The views of indoor mapping with the iPhone 14 Pro: (**a**) PIX4DCatch view; (**b**) 3D Scanner view.

Both PIX4DCatch and 3D Scanner support high-precision 3D scanning of the surrounding environment using the LiDAR sensor on the iPad Pro or iPhone 14 Pro. Both of them support the output of triangle mesh data in OBJ format, which allows us to easily process and use them for subsequent processing. During our experiments, we installed PIX4DCatch version of 1.28.1 (813) and 3D Scanner version of 2.0.17 (2) on the iPhone 14 Pro, respectively. For PIX4DCatch, we chose to skip low-quality images during the scanning process, selected normal mode for the resolution of the acquired images, set the overlap between images to 90%, and set the camera to autofocus. For 3D Scanner, we selected the advanced LiDAR scanning mode for the mapping process. The scanning confidence parameter was set to the highest level, and the maximum depth range was set at 5.0 m. The resolution of the generated data was established at 50 mm. Additionally, we did not enable the masking feature, which has the capability to mask LiDAR data based on the type of object in view. It is worth mentioning that all data acquisition and processing were performed on the iPhone 14 Pro and finally exported in OBJ format. The views of indoor mapping with PIX4DCatch and 3D Scanner are shown in Figure 6.

The schematic overview depicted in Figure 1 illustrates the evaluation process in this paper. For the collection of the experimental data, we used the HoloLens 2 and iPhone 14 Pro to scan the corridor environment, respectively. Each data collection session started from the same point, with the direction of travel for each mapping strategy shown in Figure 3. When performing manual mapping, since it is challenging to maintain complete consistency in the trajectory, speed, and pose of the device during each mapping, a set of principles was applied to maximize the consistency across each scan. These principles include walking along the middle of the corridor, maintaining a steady pace frequency, and avoiding hand jerks up and down. In order to reduce the impact of human factors, we performed five data collections for each mapping strategy. Such processing helps to improve the consistency and reliability of the data.

For the ground truth data, we used the dataset created by Schmidt et al. [68], which is point clouds collected by the terrestrial laser scanner Imager 5016 by Zoller & Fröhlich. The 5016 imager has the capability to capture up to 1 million points per second. The scanning process was executed from diverse scanner positions without the need for fixed points or targets. Then, the software Scantra (v.3.2) (Technet GmbH, Berlin, Germany) was used for registering the point clouds. The processed point clouds were exported as E57-files. In the evaluation work, we extracted the corridor area of the dataset as our ground truth.

The software CloudCompare 2.13.alpha version [69] was used for the process of data evaluation. First, we performed data preprocessing to remove redundant data unrelated to the evaluation. Then, we manually selected point pairs to align the mesh entities and point cloud entities, and performed fine registration using the iterative closest point (ICP) algorithm [70,71,72]. During the fine registration phase, we set the number of iterations to 20, the root mean square (RMS) difference to 1.0 × 10^−5^, the final overlap to 100%, and the random sampling limit to 50,000. In this process, we chose to rotate and translate the aligned data in the XYZ axis without adjusting the scale. We did not enable the removal of the farthest points, and we did not utilize cloud-to-mesh (C2M) signed distances, as our focus was on overall alignment rather than on the directionality of discrepancies.

For the accuracy evaluation, we adopt the cloud-to-cloud (C2C) Gaussian mean distance as the metric, which is the mean distance between two point clouds to measure the match accuracy between the data collected and the ground truth. Referring to the work of Hübner et al. [28], we employed the vertices of the triangle mesh data and points from the ground truth for the calculation. 

During the C2C distance computation in CloudCompare, we configured the related parameters as follows: the octree subdivision level was set to automatic for optimal balance, the maximum distance was set to default 2.25 m, and we did not split the distances into X, Y, and Z components. Additionally, we did not apply any local surface modeling. These settings were chosen to focus on the global spatial relationships within the collected triangle mesh and ground truth, without being influenced by local geometric variations or axis-specific deviations.

As for the evaluation of data completeness, our study focused on the inclusion and coverage of critical environmental features within the collected triangle mesh. We specifically examined elements such as walls, pillars, and furniture, ensuring that the mesh accurately represented the full extent of the surveyed environment.

## 3. Results

In this section, we present the results of the indoor mapping experiments with the Microsoft HoloLens 2 and iPhone 14 Pro under the four different mapping strategies. First, in Section 3.1, we present the outputs of the indoor mapping using the HoloLens 2 and iPhone 14 Pro with PIX4DCatch and 3D Scanner. Afterwards, the results of the indoor mapping accuracy are presented in Section 3.2.

**Figure 7:**
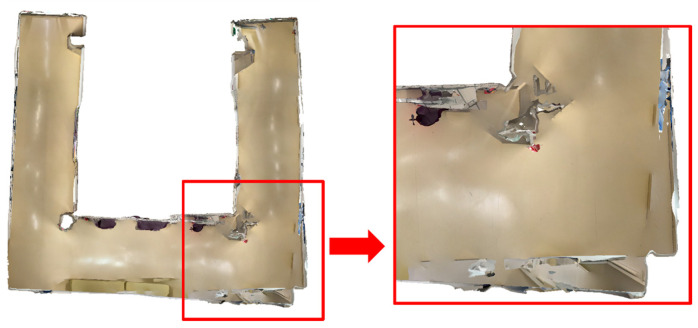
An instance of occasional significant drift that occurred when mapping with the iPhone 14 Pro under round-trip strategies. The ceiling is removed for better visibility.

It should be mentioned that when using the iPhone 14 Pro for round-trip indoor mapping, there was occasional significant drift. For instance, when using 3D Scanner for RT-LRAM strategy indoor mapping on the iPhone 14 Pro, the walls were severely misaligned, as shown in Figure 7. Occasionally, a similar situation also occurred when using PIX4Dcatch for round-trip mapping. However, no such significant drift was observed during data acquisition with the HoloLens 2. When significant drift occurred, we discarded the existing data and acquired new data using the same strategy as a replacement.

### 3.1. Indoor Mapping Outputs

**Figure 8:**
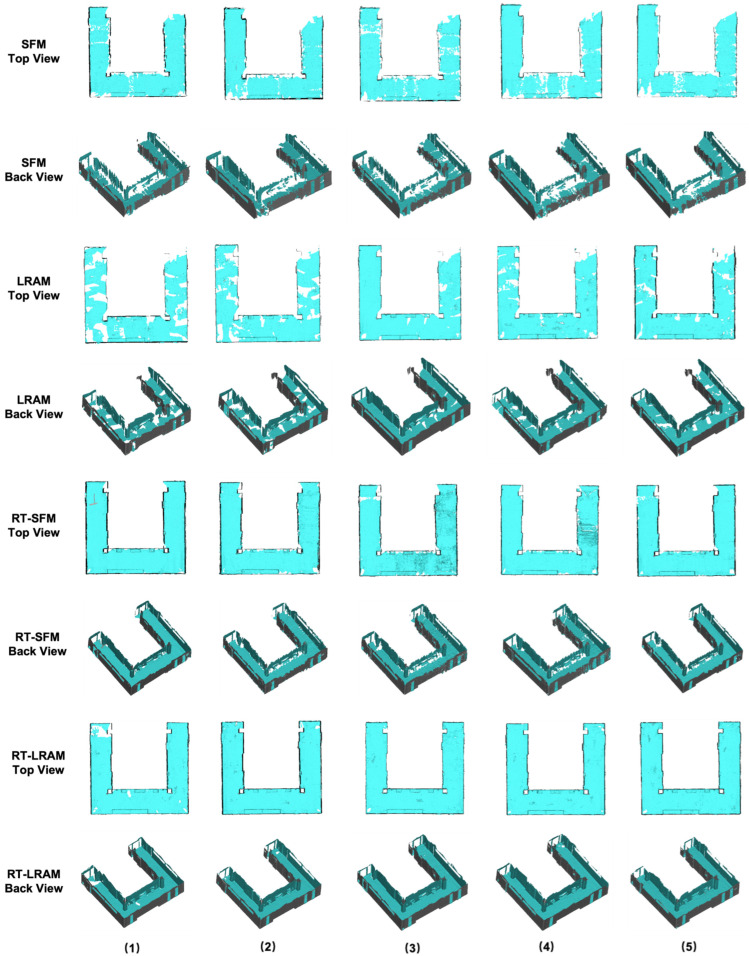
The indoor mapping outputs using the HoloLens 2 under different mapping strategies. The ceiling is removed for better visibility.

**Figure 9:**
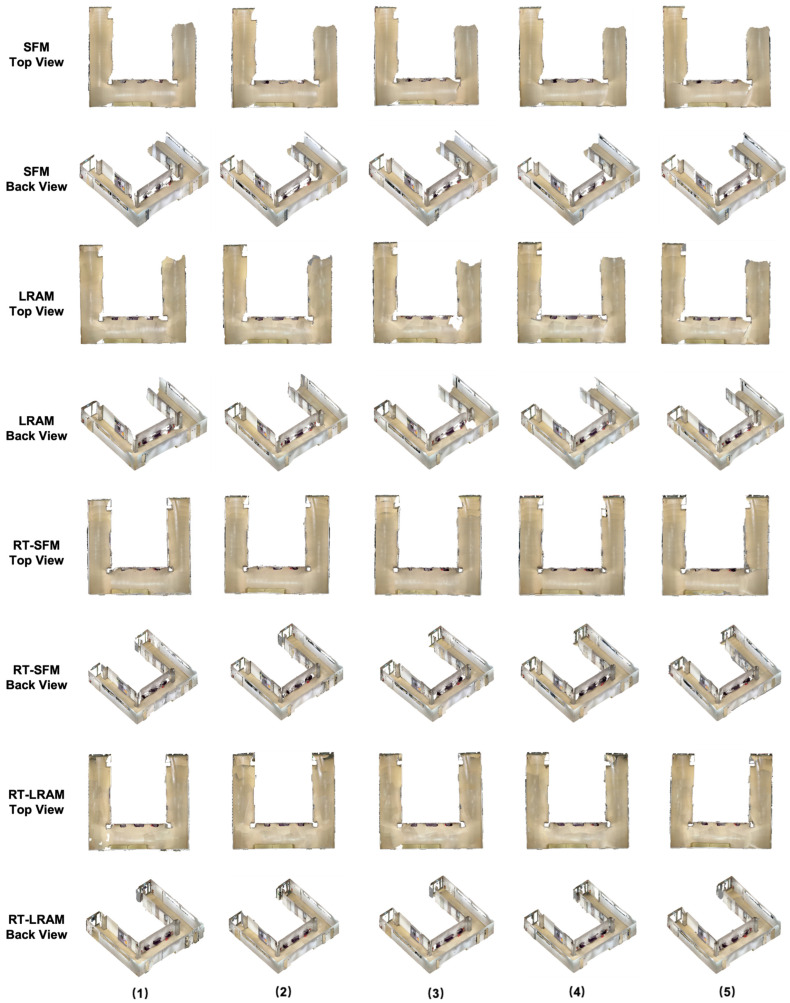
The indoor mapping outputs with PIX4DCatch on the iPhone 14 Pro under different mapping strategies. The ceiling is removed for better visibility.

**Figure 10:**
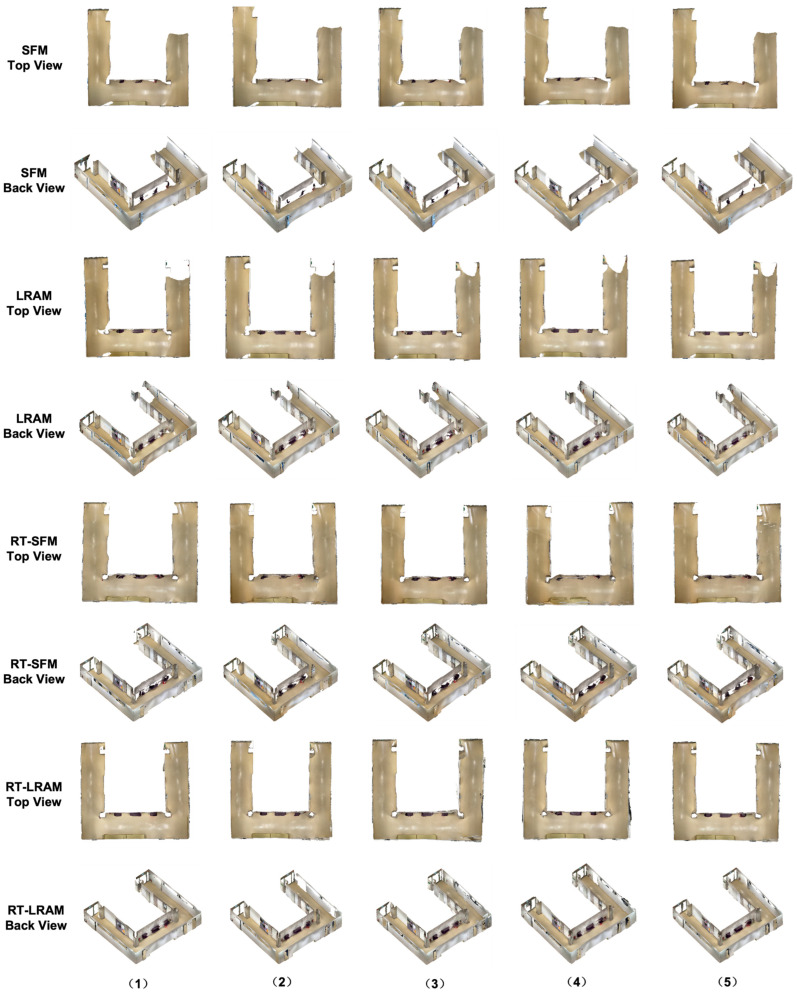
The indoor mapping outputs with 3D Scanner on the iPhone 14 Pro under different mapping strategies. The ceiling is removed for better visibility.

The outputs of indoor mapping using the HoloLens 2 and iPhone 14 Pro through different mapping strategies are shown in Figure 8, Figure 9 and Figure 10, respectively. Considering that the mapping outputs of the HoloLens 2 lack texture from images, we computed per-triangle faceted mesh normal in CloudCompare to enhance visualization. In contrast, the mapping outputs from the iPhone 14 Pro are presented as a textured triangle mesh.

To provide a more intuitive visualization for observing the performance of the HoloLens 2 and iPhone 14 Pro under the four simplified mapping strategies. We selected the corresponding five scan sequences under each strategy and presented them in order. With our top and back views, we can clearly observe the completeness of the output triangle mesh data. It should be mentioned that in our data presentation and evaluation, we mainly retained the relevant triangle mesh of the experimental environment from the output of both the HoloLens 2 and iPhone 14 Pro, the irrelevant data beyond this were carefully removed to prevent their potential impact on the evaluation results.

By comparing with the scene depicted in Figure 2, we can assess the overall performance of indoor mapping using the HoloLens 2, iPhone 14 Pro with PIX4DCatch, and iPhone 14 Pro with 3D Scanner across different strategies.

### 3.2. Indoor Mapping Accuracy 

**Figure 11:**
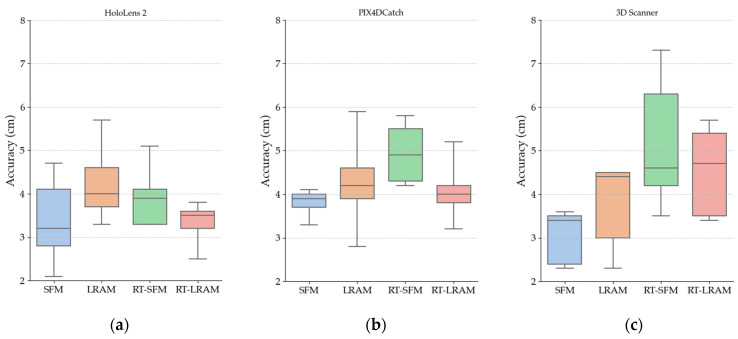
Box plot of the Gaussian mean for indoor mapping accuracy: (**a**) accuracy of the HoloLens 2; (**b**) accuracy of the iPhone 14 Pro with PIX4DCatch; (**c**) accuracy of the iPhone 14 Pro with 3D Scanner.

In this section, we present the results of the indoor mapping accuracy across the Gaussian mean of the distance between the vertices in triangle mesh data and the points in ground truth data, as shown in Table 1. For enhanced visibility, we present the Gaussian mean in Figure 11 using box plots. Moreover, we list the Gaussian standard deviation in Table 2 which is associated with the Gaussian mean.

**Figure 12:**
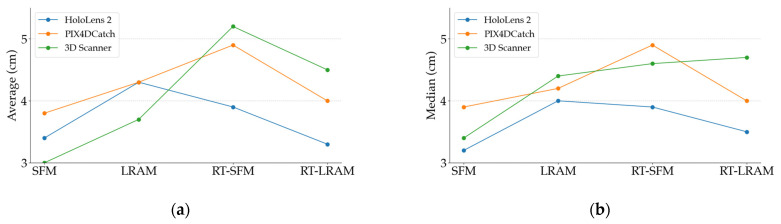
Line chart of the average and the median of the Gaussian mean for indoor mapping accuracy: (**a**) the average of the Gaussian mean; (**b**) the median of the Gaussian mean.

**Figure 13:**
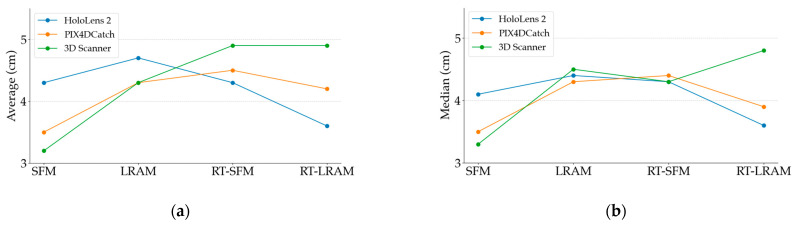
Line chart of the average and the median of the Gaussian standard deviation associated with the Gaussian mean for indoor mapping: (**a**) the average of the Gaussian standard deviation; (**b**) the median of the Gaussian standard deviation.

For a more in-depth analysis, we calculated the average of the accuracy of the five scanning sequences under each mapping strategy and presented them in line charts. At the same time, we also depicted the trend of the median variation of the accuracy of these five scanning sequences by line charts. The line charts in Figure 12 illustrate both the average and median values of the indoor mapping accuracy in Table 1. Correspondingly, Figure 13 presents the average and median values of the standard deviation for Table 2. 

In this way, the accuracy distribution of the scanning sequences and its variation under different mapping strategies can be more intuitively shown to provide strong support for the subsequent analyses.

## 4. Discussion

In the following, we discuss the indoor mapping results presented in Section 3. We first analyze the output results of indoor mapping in Section 4.1, and then discuss the results of the indoor mapping accuracy evaluation using the HoloLens 2, iPhone 14 Pro with PIX4DCatch, and iPhone 14 Pro with 3D scanner under different strategies in Section 4.2.

### 4.1. Indoor Mapping Outputs Analysis

**Figure 14:**
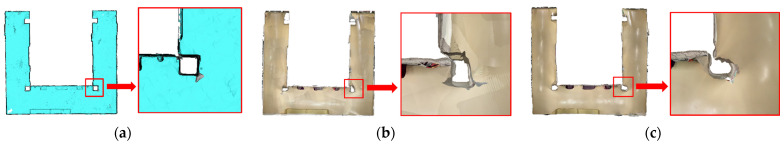
Example of pillar contours detail in the output triangle mesh: (**a**) mesh output by the HoloLens 2; (**b**) mesh output by the iPhone 14 Pro with PIX4DCatch; (**c**) mesh output by the iPhone 14 Pro with 3D Scanner.

From the triangle mesh data presented in Figure 8, Figure 9 and Figure 10, we can see that both the HoloLens 2 and iPhone 14 Pro obtained the highest data completeness using the RT-LRAM scanning strategy. Both the surrounding walls and floors have high completeness. Overall, the HoloLens 2 obtained more stable data than the iPhone 14 Pro, especially for the contours of the four pillars in the corridor. As shown in Figure 14, the contours of pillars in the triangle mesh obtained by the HoloLens 2 are more regular and uniform.

For the triangle mesh obtained with the HoloLens 2 and iPhone 14 Pro under the SFM, LRAM, and RT-SFM strategies, we found that there is a certain degree of surfaces missing on both walls and floors. Obviously, the data completeness collected under the SFM strategy is the worst, especially in the central area of the corridor where the wall is missing the most. By comparing the mapping results of the HoloLens 2 and iPhone 14 Pro, we can see that the completeness of the data captured by the iPhone 14 Pro is slightly higher than that of the HoloLens 2 under the SFM and LRAM strategies. The data obtained by the HoloLens 2 under these two mapping strategies is missing not only on the wall surface but also on the floor surface. In contrast, the missing portion of the surface of the data obtained by the iPhone 14 Pro was concentrated on the walls in the central area of the corridor. 

We speculate that the main reason for the incomplete walls with the SFM, LRAM, and RT-SFM strategies, is the presence of suspended tables and cabinets on the walls in the central area of the corridor, as shown in Figure 2. This obstruction interferes with the infrared light emitted by the HoloLens 2 and the LiDAR signal emitted by the iPhone 14 Pro, preventing the perception of depth information and the generation of a complete triangle mesh. However, the data completeness under the LRAM and RT-SFM strategies is better than SFM, suggesting that left–right alternative mapping and round-trip mapping can compensate for the effects of occlusion to some extent.

In addition, for the surfaces missing in the triangle mesh obtained by the HoloLens 2 under the SFM and LRAM strategies, particularly on floors, we speculate that the main reason is due to the low frame rate of the long-throw depth-sensing camera of the HoloLens 2. However, from the output results of RT-SFM and RT-LRAM, we can see that round-trip mapping can compensate for the low frame rate of the long-throw depth-sensing camera and make the collected data more complete.

It is important to mention that when we use the HoloLens 2 for data collection, we can export the trajectories of the HoloLens 2 and the timestamps corresponding to each pose. As a result, we can calculate the average speed at which the HoloLens 2 moved during each scan, which ranges from 0.335 ± 0.024 m per second across a total of 20 data acquisitions. Additionally, through the output files provided by PIX4DCatch and 3D Scanner, we also calculated the average speed at which the iPhone 14 Pro moved during each scan, ranging from 0.400 ± 0.036 m per second with PIX4DCatch to 0.343 ± 0.040 m per second with 3D Scanner. From the average speed, we can see that we took meticulous measures to ensure a consistent step rate for both the HoloLens 2 and the iPhone 14 Pro during each mapping process.

Nevertheless, we performed thorough mapping using the HoloLens 2 and iPhone 14 Pro, respectively. While these mapping processes are difficult to describe accurately in words, we tried to keep the scanning speed slower and scan every part of the scene until the live-mapping visualization is as good as can be. Finally, after multiple attempts, we obtained the most accurate results for the data collected in the thorough mapping mode as a reference. The outcomes were as follows: The HoloLens 2 delivered an accuracy of 3.1 cm and an average speed of 0.275 m per second. When utilized with PIX4DCatch, the iPhone 14 Pro achieved an accuracy of 3.5 cm and an average speed of 0.261 m per second. On the other hand, when working with 3D Scanner, the iPhone 14 Pro delivered an accuracy of 2.7 cm and an average speed of 0.308 m per second. It should be mentioned that in order to obtain more complete data, the movement trajectories of the devices in thorough mapping mode are round-trip mapping.

Generally, it is clear that round-trip mapping can produce a more complete data output, but during data acquisition, we found that using the iPhone 14 Pro for round-trip data acquisition using PIX4DCatch and 3D Scanner occasionally resulted in significant drift, as shown in Figure 7. However, when using the HoloLens 2 for round-trip mapping, this did not occur. We hypothesize that in the round-trip situation, the HoloLens 2 detected loop closure and corrected the device’s position accordingly and locally removed the incorrectly positioned meshes, while the built-in indoor mapping algorithms of PIX4DCatch and 3D Scanner may not consider loop closure detection, resulting in occasional significant drifts in the output triangle mesh.

### 4.2. Indoor Mapping Accuracy Evaluation

In Section 3.2, we separately calculated the accuracy of indoor mapping under four different mapping strategies for HoloLens 2 and iPhone 14 Pro. 

**Figure 15:**
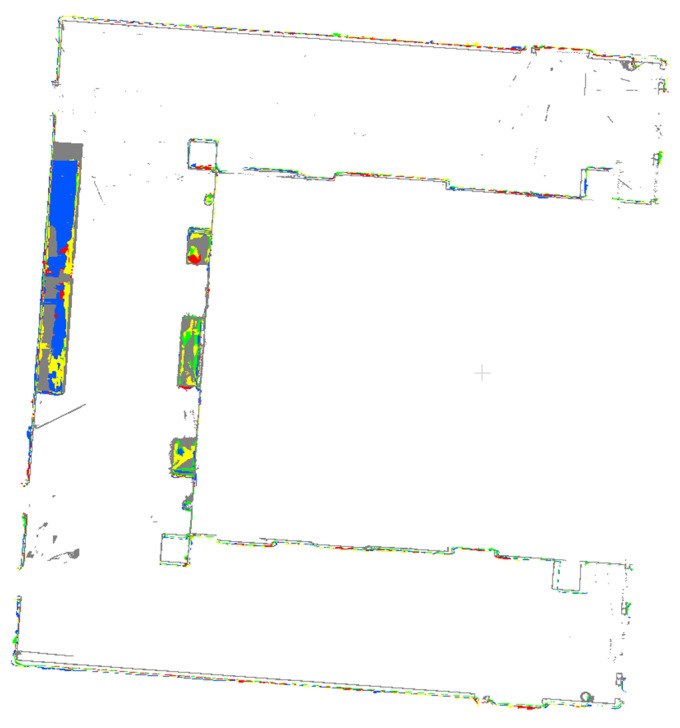
The cross section comparison of the data acquired by HoloLens 2, represented by the data with median accuracy in Table 1. Red represents data across the SFM strategy, yellow represents LRAM, blue represents RT-SFM, green represents RT-LRAM, and grey represents the ground truth data.

As shown in Figure 12, for HoloLens 2 indoor mapping accuracy, the maximum difference between the average values, as well as the maximum difference between the median values, is around 1 cm for different mapping strategies, which indicates that the indoor mapping accuracy of HoloLens 2 remains almost consistent across the four different scanning strategies. Furthermore, as observed in Figure 15, the cross section comparison of the data obtained by HoloLens 2 also highlights its exceptional stability.

Although different mapping strategies have limited impact on the indoor mapping accuracy of HoloLens 2, choosing an appropriate mapping strategy can still improve data accuracy and data completeness. By observing the indoor mapping accuracy of HoloLens 2 in Figure 12, we can see that the data accuracy obtained under the LRAM strategy is the worst compared to other mapping strategies. The data accuracy under the SFM strategy appears to be higher than that of LRAM, but the obtained triangle meshes have the worst completeness. To improve the completeness of the obtained triangle mesh, mapping strategies like LRAM, RT-SFM or RT-LRAM may be utilized. 

However, under the premise of ensuring the good completeness of the acquired triangle mesh data, the data accuracy across the RT-SFM and RT-LRAM strategies is higher than that of LRAM, especially the data accuracy under the RT-LRAM strategy is the highest in Figure 12, indicating that round-trip mapping can compensate to some extent for errors caused by drifts of the HoloLens 2 during data acquisition, as the indoor mapping algorithm of HoloLens 2 includes loop closure detection and local error correction capabilities. 

**Figure 16:**
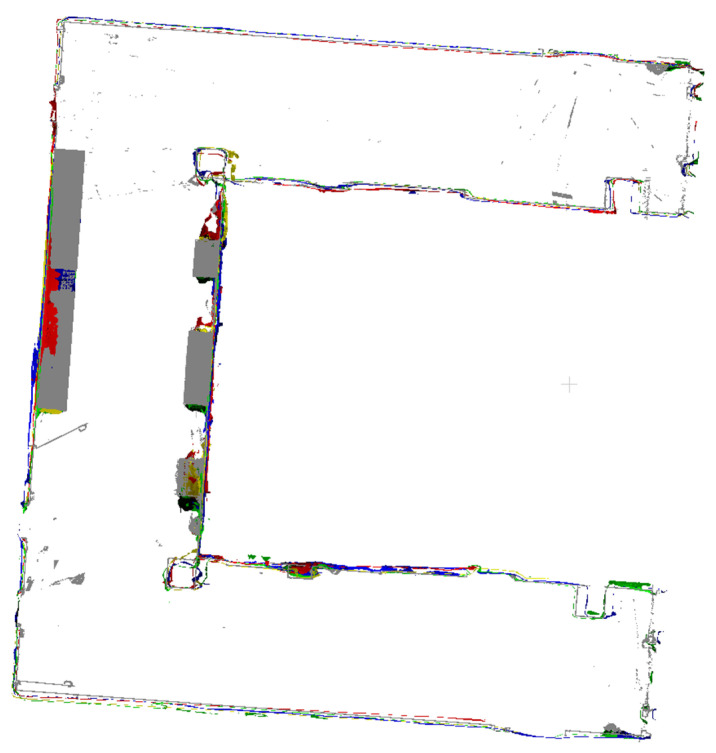
The cross section comparison of the data acquired by the iPhone 14 Pro with PIX4DCatch, represented by the data with median accuracy in Table 1. Red represents data across the SFM strategy, yellow represents LRAM, blue represents RT-SFM, green represents RT-LRAM, and grey represents the ground truth data.

**Figure 17:**
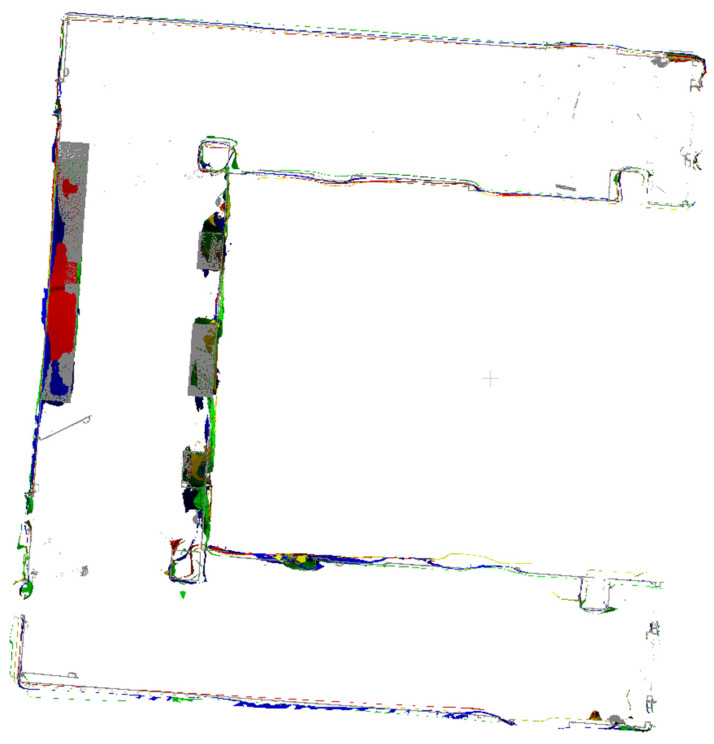
The cross section comparison of the data acquired by the iPhone 14 Pro with 3D Scanner, represented by the data with median accuracy in Table 1. Red represents data across the SFM strategy, yellow represents LRAM, blue represents RT-SFM, green represents RT-LRAM, and grey represents the ground truth data.

Similarly, by observing the indoor mapping accuracy of the iPhone 14 Pro using PIX4DCatch and 3D Scanner in Figure 12, we can see that under different mapping strategies, the fluctuation range of the indoor mapping accuracy for the iPhone 14 Pro is larger than that of the HoloLens 2. Especially with the cross section comparison in Figure 16 and Figure 17, we can clearly see that the data obtained by the iPhone 14 Pro using PIX4DCatch and 3D Scanner under different strategies is less stable than the HoloLens 2. The difference in mapping accuracy between PIX4DCatch and 3D Scanner under the same strategy is around 1cm. This suggests that when performing indoor mapping with the iPhone 14 Pro, the accuracy difference between different off-the-shelf commercial applications may not be significant, since most of them are developed based on Apple’s ARKit framework.

Furthermore, we found that the two applications generally have the same trend in data accuracy under different mapping strategies. Although there is a slight difference in the accuracy trend under RT-LRAM, the variation is only around 1cm. In combination with the occasional significant drift in the triangle mesh data during the round-trip mapping process mentioned in Section 3, we can conclude that round-trip mapping with the iPhone 14 Pro may not necessarily reduce errors in the triangle mesh data. In some instances, it might even increase the probability of noticeable drift occurrences. Therefore, we hypothesize that PIX4DCatch and 3D Scanner may not incorporate loop closure detection in their indoor mapping algorithms. Consequently, when the iPhone 14 Pro utilizes these two applications for indoor mapping, the errors may gradually accumulate over time. Despite the potential for drift with round-trip mapping, the RT-LRAM strategy is still a good choice for higher data completeness, especially suitable for medium-scale indoor spaces.

## 5. Conclusions

This study explored the often overlooked issue of the impact of user behavior on the evaluation of indoor mapping with entertainment devices, which may lead to potential errors and limitations in practical applications. Our evaluation shed light on the varying degrees of data completeness and accuracy in indoor mapping using the HoloLens 2 and iPhone 14 Pro across different strategies.

According to our prior knowledge, we first defined four simplified mapping strategies based on different user behaviors. Subsequently, we collected triangle mesh data under each strategy using the HoloLens 2 and iPhone 14 Pro. For indoor mapping with the HoloLens 2, we collected triangle mesh data using an in-house developed mapping script based on an open-source library, and for indoor mapping with the iPhone 14 Pro, we used two different off-the-shelf applications, PIX4DCatch and 3D Scanner, for triangle mesh data acquisition. In this work, we used the point clouds collected by an industrial-grade terrestrial laser scanner as the ground truth, and finally carried out the data accuracy assessment work with CloudCompare.

The study shows that the HoloLens 2 performs better in indoor mapping compared to the iPhone 14 Pro. The triangle mesh accuracy collected by the HoloLens 2 is more stable and less affected by different strategies. Under the RT-LRAM strategy, the HoloLens 2 achieves the best data results, which effectively compensates for the surface missing on walls and floors caused by furniture occlusion and the low frame rate of long-throw depth-sensing camera of the HoloLens 2. In addition, the round-trip mapping strategy slightly reduces the mapping errors of the HoloLens 2. For the iPhone 14 Pro, triangle mesh data acquired using PIX4DCatch and 3D Scanner showed similar indoor mapping accuracy for the same strategy, with a difference of approximately 1 cm. However, the triangle mesh data collected by the round-trip mapping strategy using PIX4DCatch and 3D scanner is more prone to drift and error accumulation. In terms of mapping completeness, the iPhone 14 Pro is more efficient than the HoloLens 2 and is more likely to scan quickly to get a more complete triangle mesh with the same mapping strategy.

Therefore, in practical applications, which entertainment device to choose for indoor mapping needs to be weighed against specific needs and scenarios. If the requirements for triangle mesh accuracy and stability are high, the HoloLens 2 may be more suitable; while if more attention is paid to mapping completeness and efficiency, the iPhone 14 Pro may be more beneficial. At the same time, to address the limitations of different devices, technical optimization and algorithmic improvements can be made to improve their mapping performance to meet the needs of different scenarios.

In this work, we only evaluated the indoor mapping accuracy under four simplified strategies utilizing the HoloLens 2 and iPhone 14 Pro. However, real-world applications may need to consider a wider range of factors, including various spatial environments, object materials, and reflective properties. Our results are mainly relevant to the devices mentioned above. We are also aware of possible differences in software and hardware between generations, as well as the challenges posed by more complex indoor environments. In future studies, we will conduct more in-depth investigations to address these challenges and improve the applicability of our findings.

## Figures and Tables

**Table 1:** The Gaussian mean for indoor mapping accuracy. Indoor mapping using the HoloLens 2, iPhone 14 Pro with PIX4DCatch, and iPhone 14 Pro with 3D Scanner under SFM, LRAM, RT-SFM, and RT-LRAM (values in cm).

Devices	Sequence	SFM	LRAM	RT-SFM	RT-LRAM
HoloLens 2	1	2.1	4.6	3.9 *	3.2
	2	2.8	5.7	4.1	3.5 *
	3	4.7	4.0 *	3.3	2.5
	4	3.2 *	3.7	5.1	3.6
	5	4.1	3.3	3.3	3.8
	**Average**	**3.4**	**4.3**	**3.9**	**3.3**
iPhone 14 Pro with PIX4Dcatch	1	3.3	3.9	4.2	4.2
	2	4.0	5.9	4.9 *	3.8
	3	3.9 *	2.8	4.3	3.2
	4	4.1	4.2 *	5.5	4.0 *
	5	3.7	4.6	5.8	5.2
	**Average**	**3.8**	**4.3**	**4.9**	**4.1**
iPhone 14 Pro with 3D Scanner	1	3.4 *	4.5	3.5	3.5
	2	2.3	2.3	7.3	4.7 *
	3	2.4	4.4 *	4.2	5.7
	4	3.6	4.5	6.3	3.4
	5	3.5	3.0	4.6 *	5.4
	**Average**	**3.0**	**3.7**	**5.2**	**4.5**

* The median of the Gaussian mean in each of the 5 sequences.

**Table 2:** The Gaussian standard deviation associated with the Gaussian mean for indoor mapping using the HoloLens 2, iPhone 14 Pro with PIX4DCatch, and iPhone 14 Pro with 3D Scanner under SFM, LRAM, RT-SFM, and RT-LRAM (values in cm).

Devices	Sequence	SFM	LRAM	RT-SFM	RT-LRAM
HoloLens 2	1	3.4	5.5	3.9	3.5
	2	4.0	5.5	4.0	4.2
	3	5.3	4.4 *	4.3 *	3.0
	4	4.1 *	3.9	5.0	3.6 *
	5	4.8	4.1	4.3	3.8
	**Average**	**4.3**	**4.7**	**4.3**	**3.6**
iPhone 14 Pro with PIX4Dcatch	1	3.2	4.3 *	4.2	4.8
	2	3.5 *	5.2	4.4 *	3.5
	3	3.5	3.7	4.3	3.8
	4	3.6	4.0	4.5	3.9 *
	5	3.6	4.3	5.2	4.9
	**Average**	**3.5**	**4.3**	**4.5**	**4.2**
iPhone 14 Pro with 3D Scanner	1	3.7	4.9	4.0	3.8
	2	2.4	3.6	6.0	4.8 *
	3	2.6	4.9	4.3 *	6.5
	4	3.3 *	4.5 *	5.9	4.2
	5	3.9	3.8	4.2	5.1
	**Average**	**3.2**	**4.3**	**4.9**	**4.9**

* The median of the Gaussian standard deviation in each of the five sequences.

## Data Availability

The data presented in this study are available on request from the corresponding author. The data are not publicly available due to privacy.
